# The Investigation of Changes in Bacterial Community of Pasteurized Milk during Cold Storage

**DOI:** 10.3390/foods13030451

**Published:** 2024-01-31

**Authors:** Xinyi Lan, Shuyan Wu, Qijing Du, Li Min

**Affiliations:** 1 College of Animal Science and Technology, Hunan Agricultural University, Changsha 410128, China; lanxinyi195@163.com; 2 Hopkirk Research Institute, AgResearch Ltd., Palmerston North 4442, New Zealand; shuyan.wu@agresearch.co.nz; 3 Grasslands Research Centre, AgResearch Ltd., Palmerston North 4472, New Zealand; qijingdu@163.com; 4 College of Food Science and Engineering, Qingdao Agricultural University, Qingdao 266109, China; 5 Ministry of Agriculture Key Laboratory of Animal Nutrition and Feed Science in South China, Institute of Animal Science, Guangdong Academy of Agricultural Sciences, Guangzhou 510640, China

**Keywords:** microorganism, pasteurized milk, cold storage, *Bacillus*, *Streptococcus*

## Abstract

The quality of pasteurized milk is commonly assessed through microbiological analysis, with variations in storage conditions significantly impacting the suppression of bacterial growth throughout the milk’s shelf life. This study investigated the dynamics of total bacterial counts (TBCs) and bacterial community shifts in milk that underwent pasteurization at 80 °C for 15 s. The milk was subsequently stored at 4 °C for varying intervals of 1, 4, 7, 10, 13, and 16 days. Culture-based testing revealed a significant TBC increase during the storage period spanning 1 to 16 days (up to −log10 4.2 CFU/mL at day 16). The TBC in pasteurized milk exhibited accelerated microbial growth from day 13 onwards, ultimately peaking on day 16. *Bacillus* was detected through 16S rRNA identification. Principal component analysis demonstrated a significant impact of storage time on bacterial communities in pasteurized milk. Analysis of bacterial diversity revealed a negative correlation between the Shannon index and the duration of pasteurized milk storage. Using high-throughput sequencing, *Streptococcus* and *Acinetobacter* were detected as prevalent bacterial genera, with *Streptococcus dysgalactiae* and *Streptococcus uberis* showing as dominant taxa. The presence of *Streptococcus dysgalactiae* and *Streptococcus uberis* in pasteurized milk might be attributed to the initial contamination from raw milk with mastitis. This study offers new evidence of the prevalence of bacterial community in pasteurized milk, thereby adding value to the enhancement of quality control and the development of strategies for reducing microbial risks.

## 1. Introduction

Dairy products are widely recognized as essential components of a healthy diet [1]. Raw milk contains a diverse spectrum of bacterial populations including beneficial lactic acid bacteria that contribute to milk processing as well as spore-forming and psychrotrophic bacteria associated with spoilage and potential health concerns. Microbial contamination within the dairy chain can manifest at various stages, spanning from on-farm origins, during transportation, to processing facilities. Consequently, both the raw and pasteurized milk microbiota composition is profoundly shaped by numerous factors including farm management practices, seasonal variations, hygienic protocols, and storage conditions spanning the entire value chain [2]. Pasteurization, a thermal treatment to eliminate pathogenic microorganisms, holds a critical role in extending milk shelf life and enhancing product quality [3,4]. In many countries, especially in the United States, consumers prefer high-temperature short-time (HTST) fluid milk to ultra-high temperature (UHT) milk with a longer shelf life. This, in turn, can lead to higher sales of dairy products and contribute to the reduction in food waste [5]. The dairy industry continues to face a significant challenge in the production of pasteurized milk with an extended shelf life and enhanced quality throughout its shelf life.

Maintaining effective temperature control throughout the entire process, from milking to storage in the raw milk bulk tank, is of importance [6]. In some cases, cold storage is not consistently maintained for consumer refrigerators, resulting in an increase in the susceptibility to spoilage and the proliferation of potential pathogenic microorganisms in stored dairy products [2]. The proper storage temperature of pasteurized milk is vital for minimizing bacterial growth [7]. Earlier studies demonstrated the risks associated with insufficient refrigeration conditions, which could lead to spoilage and potential pathogen growth during storage [8]. Current studies have primarily focused on the storage temperature of raw milk and UHT milk [9,10]. A substantial proliferation of bacteria might occur during the storage time in raw milk [9]. During the storage of UHT milk at cold storage (3 °C), notable alterations occur in the protein structure, consequently impacting the quality of the product. These changes become more pronounced under high-temperature storage conditions [10]. Other studies have investigated various approaches to extend the shelf-life of HTST fluid milk [6]. A recent study indicated that storage temperature has a substantially larger effect on fluid milk shelf life than HTST and suggests that abuse temperatures (e.g., storage at 10 °C) allow for the growth of *Bacillus* species that do not grow at lower temperatures [11]. Limited evidence exists regarding the bacterial variation of pasteurized milk during cold storage. Therefore, understanding the change in bacterial communities during storage could provide new insights into the degradation of pasteurized milk. 

Both microbial culture and advanced high-throughput sequencing methods have been widely employed to identify and characterize the microorganisms present in milk [2]. Culture methods offer reliability in microbial community analysis, but they often come with time-consuming and labor-intensive processes that might not fully capture the diverse complexity of microbial communities. Polymerase chain reaction (PCR) cloning and the sequencing of the 16S ribosomal RNA gene (rRNA) are also popularly applied as the standard methods [12]. The culture method is unable to detect some challenging-to-culture strains or those in a viable but nonculturable (VBNC) state. High-throughput sequencing provides a more accurate method for obtaining information about microbial colonies, enabling the detection of low-abundance microorganisms [3]. Furthermore, a more advanced high-throughput sequencing method can generate far more reads than the culture method and facilitate the discovery of even greater bacteria diversity [13]. This study aimed to investigate alterations in the bacterial community and quality attributes of pasteurized milk during storage, using these methods to identify bacteria in milk samples stored at low temperatures. The research outcomes provide new insights into potential bacterial risks in pasteurized milk during cold storage, while also gaining a better understanding of variations in pasteurization safety.

## 2. Materials and Methods

### 2.1. Collection and Treatment of Milk Samples 

In September 2022, a total of five factories were chosen from various regions in Henan, China, to collect pasteurized bovine milk. In each commercial dairy industrial factory, three bottles of pasteurized milk (80 °C/15 s) were collected on five separate dates. The collected milk was promptly transferred into 200 mL sterile bottles. All samples were transported to the laboratory and then stored at 4 °C for up to 16 days. The pasteurized 95 milk samples were taken for analysis at 1, 4, 7, 10, 13, and 16 days of storage.

### 2.2. Culture-Based Microbial Identification 

TBC was immediately detected upon arrival at the laboratory, representing the initial value (day 0). Milk samples were subjected to total bacterial count (TBC) detection using culturing methods in accordance with the National Standards of the Republic of China [14]. Briefly, 25 mL of each milk sample was homogenized in 225 mL diluent solution (0.85% NaCl). A series of 10-fold dilutions were prepared and each diluent was spread on Plate Count Agar (PCA, Beijing Land Bridge Technology Co. Ltd., Beijing, China). The plates were incubated at 37 °C for 48 h. The results are expressed as decimal logarithms of colony-forming units per milliliter (log10 CFU/mL). Each sample and its diluents were plate-counted in triplicate. 

### 2.3. DNA Extraction and Pyrosequencing 16S rRNA

Fifty bacterial colonies were selected from the cultured plates for DNA extraction and sequencing. The DNA of the bacterial colony was isolated using the PowerFood Microbial DNA Extraction Kit (MO BIO Laboratories, Carlsbad, CA, USA) and stored at −20 °C prior to PCR. Extracted DNA was assessed by agarose gel (1%) electrophoresis and quantified using a Nanodrop spectrometer (Thermo Scientific, Waltham, MA, USA). DNA was diluted to a concentration of 50 ng/μL and used as a template for amplification in the following PCRs. The 16S rRNA gene fragment was amplified by polymerase chain reaction with a pair of universal primers 27F (5′-AGAGTTTGATCMTGGCTCAG-3′) and 1492R (5′-TACGGYTACCTTGTTACGACTT-3′). PCR amplification began with a 5 min denaturing step at 94 °C, followed by 30 cycles at 94 °C for 30 s, 50 °C for 30 s, and 72 °C for 30 s; extension was achieved at 72 °C for 15 min. The purified PCR products were sequenced by ABI 3730XL (ThermoFisher, Waltham, MA, USA). For strain identification, the 16S rRNA gene sequences of the isolates were blasted against the NCBI nr database. 

### 2.4. The Examination of Bacterial Communities by High-Throughput Sequencing Analysis 

Total DNA was isolated from 2 mL of pasteurized milk samples that were stored for 16 days using the PowerFood Microbial DNA Extraction Kit (MO BIO Laboratories, Carlsbad, CA) and stored at −20 °C prior to PCR. The DNA extraction and qualification procedures were conducted following previously described methods.

The 16S rRNA gene fragment was amplified by polymerase chain reaction with a pair of universal primers 341F (5′-CCTAYGGGRBGCASCAG-3′) and 806R (5′-GGACTACNNGGGTATCTAAT-3′). PCR amplification began with a 5 min denaturing step at 94 °C, followed by 30 cycles at 94 °C for 30 s, 50 °C for 30 s, and 72 °C for 30 s; extension was achieved at 72 °C for 15 min [15]. PCR amplicons were extracted from 2% agarose gels and purified using the AxyPrep DNA Gel Extraction Kit (Axygen Biosciences, Union, CA, USA) according to the manufacturer’s instructions and quantified using QuantiFluor-ST (Promega US, Madison, WI, USA). Purified amplicons were pooled in equimolar and paired-end sequenced on an Illumina MiSeq platform according to the standard protocols.

Operational taxonomic units (OTUs) were generated by clustering at 97% sequence identity using the UCLUST algorithm in QIIME 1.9 [16,17]. The OTUs were further assigned to taxa using the RDP classifier [18]. The Simpson, Shannon, Chao1, and PD whole tree index were calculated for each sample in QIIME 1.9 [2]. The weighted UniFrac distance was used for principal coordinate analysis (PCoA) [19]. 

### 2.5. Statistical Analysis 

Data are expressed as the mean standard deviation (SD) of three replicates. Significant differences between the means of parameters were calculated with the Duncan’s multiple-range test using SPSS 17.0 software (SPSS, Inc., Chicago, IL, USA). *p* < 0.05 was considered statistically significant.

## 3. Results

### 3.1. Total Bacterial Counts in Pasteurized Milk 

**Figure 1:**
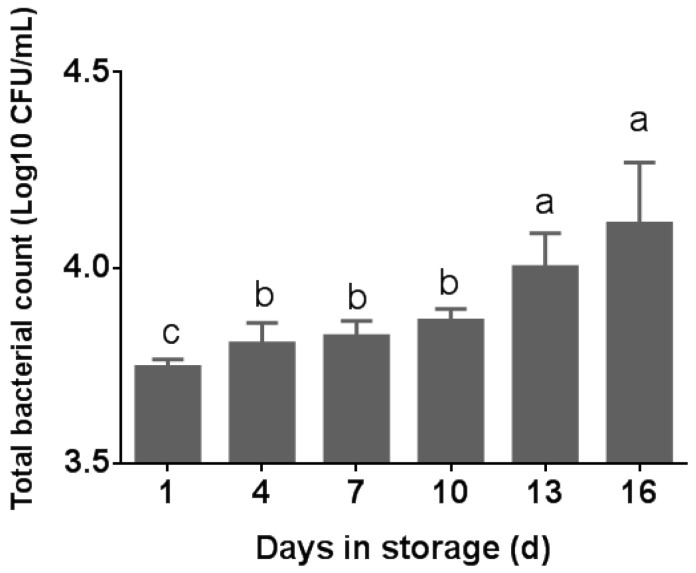
TBC in pasteurized milk samples during the storage time. Different superscripts in the bar chart indicate significant differences (*p* < 0.05).

The total bacterial count (TBC) was assessed in five pasteurized milk samples at various storage times. As depicted in Figure 1, there was a statistically significant difference in the total bacterial count (TBC) in pasteurized milk observed across different storage times (*p* < 0.05). The TBC in pasteurized milk was 1.3 log10 CFU/mL at the sampled day, which is below the standard limit. As per the China National Food Safety Standard, a maximum of 4.2 log10 CFU/mL is deemed acceptable for pasteurized milk [20]. The TBC of the samples grew rapidly from day 13, reaching the highest on day 16 (*p* < 0.05) (Figure 1). From day 4 to day 10, the TBC did not change substantially (*p* > 0.05). As shown in Figure 1, all of the milk samples we sampled did not exceed the standard. A positive correlation between the TBC and storage time of pasteurized milk at 4 °C was observed. All 50 selected bacterial colonies were identified as *Bacillus* using 16S rRNA PCR sequencing. 

### 3.2. Bacterial Diversity

**Figure 2:**
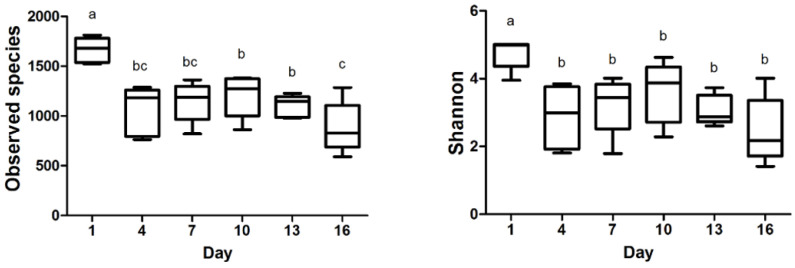
Richness and diversity indices at different storage times from pasteurized milk. Different superscripts in the bar chart indicate significant differences (*p* < 0.05).

The observed species and Shannon index data are presented in Figure 2, revealing the significant differences during storage time. Subsequently, the observed species in milk samples reached their lowest point on day 16. Between day 4 and day 16, there were no substantial fluctuations observed in the Shannon index, indicating a relatively stable microbial diversity during this timeframe. Our investigation demonstrated that the bacterial communities in milk samples displayed higher diversity values during the initial storage phase. Furthermore, the negative correlation between the Shannon index and the storage time of pasteurized milk implied a gradual decline in bacterial diversity as the storage duration increased. These findings are valuable evidence for comprehending the dynamics of bacterial communities in pasteurized milk during storage.

### 3.3. Effect of Storage Time on the Bacterial Communities in Pasteurized Milk

**Figure 3:**
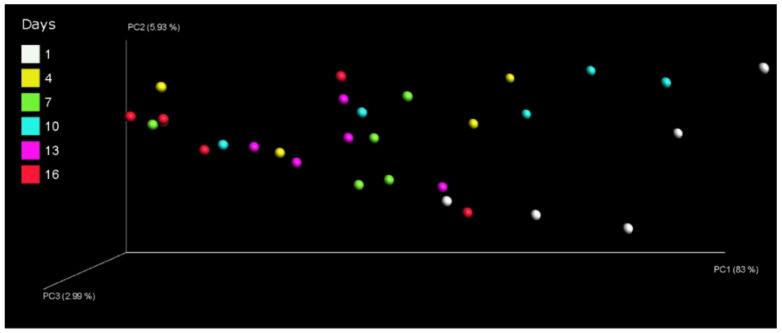
Principal coordinates analysis plot of microbial data for pasteurized milk samples during storage.

The composition of the bacterial communities of pasteurized milk during storage at 4 °C was assessed by principal coordinate analysis. The analysis of Bray–Curtis distances effectively differentiated between the various milk samples. The principal coordinates 1, 2, and 3 explained 83%, 5.93%, and 2.99% of the variation, respectively. It demonstrated a significant shift in the bacterial composition between day 1 and day 16 (Figure 3). The results suggest that storage time significantly influenced the changes in bacterial communities within pasteurized milk. 

### 3.4. The Composition of Bacterial Community 

**Figure 4:**
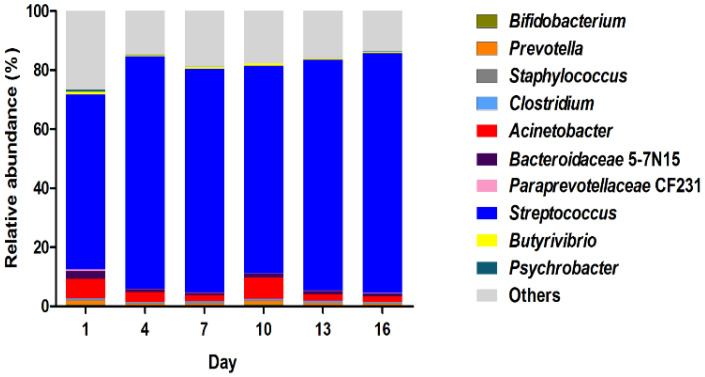
Relative abundance of top 10 most abundant bacteria at genus level in pasteurized milk samples during storage.

Bacterial diversity of the samples was evaluated, and the relative abundance was characterized at the genus level (Figure 4). During the storage period, two genera were predominant in the microbiota. *Streptococcus* emerged as the major genus, constituting 90% of all OTUs. *Acinetobacter* was the second most dominant genus, displaying variable abundance as the storage progressed. The combined abundance of other undetermined genera was lower than that of dominant *Streptococcus* but greater than *Acinetobacter*, showing a decreasing trend from day 1 to day 16. While *Streptococcus* and *Acinetobacter* were identified as the dominant components of the microbiota, our results revealed a fluctuating abundance of *Acinetobacter*, with a notable increase at day 10. These findings from our study highlight the dynamic nature of bacterial composition changes during the storage of pasteurized milk.

**Figure 5:**
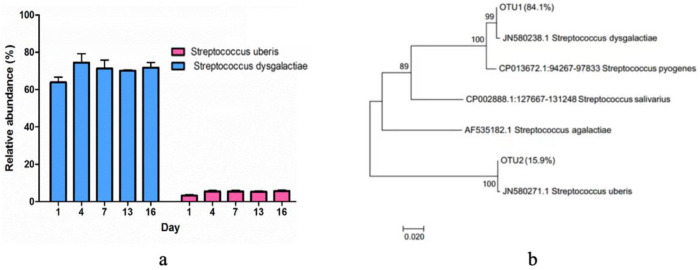
*Streptococcus* isolated from pasteurized milk samples during storage. (**a**) The relative abundance of *Streptococcus uberis* and *Streptococcus dysgalactiae* during storage. (**b**) The evolutionary history dendrogram of *Streptococcus* isolates.

Regarding the dominant *Streptococcus* group, the specific abundances of *Streptococcus uberis* and *Streptococcus dysgalactiae* were further elucidated (Figure 5a). The evolutionary history dendrogram of *Streptococcus* isolates was constructed, illustrating their relationships with reference strains and other isolated species. (Figure 5b). The findings revealed the clustering of associated taxa, where *Streptococcus dysgalactiae* represented 84.1%, and *Streptococcus uberis* constituted 15.9%. The reference strains were annotated with their corresponding GenBank accession numbers and species names, enabling precise identification and classification of the strains in the dendrogram.

## 4. Discussion

### 4.1. Effects of Storage on Bacteria in Pasteurized Milk

Raw milk and properly pasteurized milk of high quality usually exhibit low total bacterial counts (TBCs) [14]. The TBC serves as an effective indicator of the quality of both raw milk and pasteurized milk throughout the processing stages. The elevated level of microbial contamination observed in pasteurized milk can be attributed to insufficient cooling and inadequate storage conditions [21]. In this study, the TBC in pasteurized milk showed an increase during storage at 4 °C. Porcellato et al. [2] reported a similar impact of storage time and temperature on both pasteurized full-fat milk and pasteurized carton milk when stored at 8 °C, but this effect was not observed in milk samples stored at 4 °C. Liu et al. [9] also found that the microbiota remained stable with constant bacterial populations when stored at 4 °C. Another study indicated that the TBC remained relatively stable during the initial 14 days for pasteurized milk (heated at 75 °C, 85 °C, and 90 °C) stored at 3 °C, suggesting that lower storage temperatures could delay the onset of microbial and chemical changes [11]. These varied research findings could potentially be linked to factors such as the initial quality of the raw milk, variations in pasteurization procedures, and the specific conditions of low-temperature milk storage [22]. Additionally, post-pasteurization contamination (PPC) with Gram-negative bacteria is another factor leading to spoilage and reduced shelf life in high-temperature, short-time pasteurized milk. Existing evidence shows that approximately 50% of HTST-pasteurized fluid milk spoilage is due to PPC [20]. Psychrotolerant Gram-negative bacteria originating from PPC can grow in refrigerated fluid milk [23]. Utilizing raw milk with shortened storage duration can help to prevent bacterial proliferation and subsequent contamination in pasteurized milk stored at 4 °C.

In this study, the detection of *Bacillus* bacteria in the milk samples using the culture method aligns with many other studies demonstrating the prevalence of *Bacillus* in pasteurized milk. Zhai et al. [24] isolated 114 *Bacillus* strains from 133 pasteurized milk samples. Studies conducted by Gao et al. [25] and Zhao et al. [26] in China as well as by Porcellato et al. [27] in Norway reported *B. cereus* contamination rates of 27%, 12%, and 11.5%, respectively. The contamination could even be found in milk samples applied with thermal treatment at a temperature of 85 °C for a duration of 10 min, which exceeds the parameters currently employed in commercial pasteurization processes in China [24]. A current survey showed that 41% of the pasteurized milk exhibited *Bacillus* spp. under refrigerated storage (6 °C) for 21 days [28]. *Bacillus*, possessing enhanced heat resistance, exhibits resilience against pasteurization and can undergo vegetative growth during refrigerated storage due to its capacity to thrive at lower temperatures such as 6 °C or below [29]. This pathogen can lead to gastrointestinal ailments including gastric diseases, vomiting, diarrhea, and, in severe cases, fatalities. It has been reported to exhibit a high incidence of contamination in all sorts of dairy products [26]. Considering that the D-value for spores of *Bacillus* strains is near 15 min at 90 °C [29], the heating condition of 85 °C for 15 s used in this study was insufficient to inactivate *Bacillus* spores. This could lead to their persistence during storage at 4 °C for 16 days, as observed in our samples. It is necessary to consider the detection of *Bacillus* strains in raw milk, which could exist in raw milk and persist until pasteurization or induced to regrow during pasteurization or storage. These results underscore the importance of effective microbiological control measures for *Bacillus* in pasteurized milk during storage at 4 °C. 

### 4.2. Effects of Storage on the Bacterial Community Diversity of Pasteurized Milk

In this research, microbial diversity in pasteurized milk was evaluated using high-throughput sequencing of the V3 and V4 regions of the 16S rRNA gene. The influence of storage time on the milk bacterial community was addressed. Detected species and Shannon were employed to determine the alpha diversity of the microbiome in the pasteurized milk. The findings indicated that both the observed species and Shannon index values were significantly higher on day 1 in comparison to the later storage periods. This suggests that the abundance and diversity of bacterial species were most prominent during the initial storage phase. The alpha diversity of the microbiome in our samples became lower as the storage time progressed. A similar result was also reported by Liu [9], who observed fluctuations in the microbial composition of HTST milk at room temperature storage, displaying a decreasing alpha diversity from 4 h to 24 h. A loss of diversity could also be seen in low-quality milk [30,31]. The current findings suggested a decreased diversity during storage, implying that a subset of bacteria became dominant in samples [30].

The impact of different storage days on the bacterial community in pasteurized milk was presented in the PCoA (shown in Figure 3). The correlation plots of PC1 (Figure 3) showed a distance between samples of day 1 (on the right) and of day 16 (on the left), indicating significant variations in bacterial communities within pasteurized milk throughout the storage period. In the PC1 plot, the milk samples from day 1 and day 10 exhibited clustering, while the samples from day 13 and day 16 clustered in both the PC1 and PC2 plots. This suggests that there were negligible differences in bacterial community composition between the samples from day 1 and day 10 as well as between the samples from day 13 and day 16. However, the plot demonstrated a noticeable distance between the samples from day 1 and those from day 9. Xue et al. detected differences in the milk microbiota between pre-HTST pasteurized milk and post-HTST pasteurized milk stored for 10 days [32]. The growth of psychrotrophic species (within the genus *Pseudomonas*) [33] could be the major contributors to affect the PC1 and PC2 of the pasteurized milk samples.

### 4.3. The Bacterial Community Composition in Pasteurized Milk during the Storage

Our results revealed that *Streptococcus* was the dominant genus in milk samples during storage, while *Acinetobacter* was the second major genus. A previous survey showed that the prevalence of *Streptococcus* in 205 pasteurized milk from fresh milk bars was 1.5% in China [34]. In an Australian survey, *Streptococcus uberis*, *Streptococcus dysgalactiae*, and *Streptococcus agalactiae* were identified at proportions of 44%, 28%, and 22%, respectively [35]. *Streptococcus. thermophilus* was identified in soy milk and cow milk; the exopolysaccharides produced by *Streptococcus. thermophilus* induced a significant reduction in IL-6 and IL-8 expressions in intestinal cells [36]. *Pseudomonas* and *Acinetobacter* were the genera with the highest relative abundances in the bulk tank samples [37] and pasteurized milk [2]. A recent Swedish study identified *Pseudomonas*, *Streptococcus*, *Acinetobacter*, and *Staphylococcus* as the top abundant bacterial genera in tank milk [38]. The variation in bacterial community composition in raw milk could be attributed to the core microbiota present in pasteurized milk.

In this study, there was no significant difference in the dominant genera of pasteurized milk during 16 days of storage. This finding is consistent with a similar study that investigated milk microbiota over a 12-day storage period at temperatures of 5 °C and 8 °C [39]. Bacteria can remain active after pasteurization during prolonged storage. For instance, microbial interactions between probiotic strains and *Streptococcus thermophilus* improved the viability of microorganisms in fermented milk during a 21-day storage period at 4 °C [40]. Therefore, effective supervision and control measures for *Streptococcus* are necessary to manage microbial contamination in pasteurized milk. *Streptococcus* spp. was identified in all pasteurized milk samples, aligning with its categorization as a constituent of the core microbiota in pasteurized milk. Our findings are consistent with a previous study that reported *Streptococcus* spp. as one of three potential pathogens in pasteurized milk between the northern and the western regions of China [34]. *Acinetobacter* spp. are commonly found in bulk milk. The previous investigation found that 89.4% of *Acinetobacter* isolates was identified in raw milk [41]. 

*Streptococcus* has a significantly detrimental effect on milk flavor and is considered a key indicator of microbial contamination in pasteurized milk [3]. *Streptococcus* are major mastitis pathogens, along with *Staphylococcus aureus* and coliforms [42]. Thermophilic *Streptococcal* bacteria have exhibited resilience to the pasteurization process and the capacity to form biofilms in dairy equipment [2]. The analysis based on functional genes identified the presence of *Streptococcus dysgalactiae* and *Streptococcus uberis* in the pasteurized milk samples. *Streptococcus dysgalactiae* carries multiple virulence determinants and resistance genes, and can cause mastitis [43]. *Streptococcus dysgalactiae* infections can occur via several routes of transmission and may persist on dairy farms for more than one year [44]. *Streptococcus dysgalactiae* and *Streptococcus uberis* likely presented in pasteurized milk due to the fact the raw milk originates from cows suffering from mastitis, and these bacteria exist in the raw milk. Therefore, the prevention and treatment of mastitis in dairy herds are crucial for enhancing the safety of dairy products. Assessing the clinical pathogenicity of *Streptococcus* spp. based on its contagiousness revealed substantial heterogeneity among different strains [44]. Additionally, the prevalence of subclinical and clinical infections and clinical manifestations collectively suggest that the virulence of *Streptococcus dysgalactiae* in bovine mastitis is relatively lower compared to that of *Streptococcus uberi* [44]. *Streptococcus uberis*, one of the most common pathogens isolated from clinical mastitis, is prevalent in farming environments. It is regarded as a main causative agent of mastitis, leading to significant financial losses annually within the dairy industry [45]. *Streptococcus uberis* is characterized by the presence of a distinctive membrane-bound protein referred to as the surface adhesion molecule. This protein assumes a pivotal role in facilitating the adherence of *Streptococcus uberis* to bovine mammary epithelial cells through the interaction with bovine lactoferrin [46]. In this study, the detection of *Streptococcus dysgalactiae* and *Streptococcus uberis* in pasteurized milk suggests a potential association with raw milk sourced from cows with mastitis. As the pathogenicity of this bacterium is largely attributed to their ability to encode and produce numerous virulence factors [47], effective measures should be taken for controlling *Streptococcus dysgalactiae* and *Streptococcus uberi* in dairy farms.

## 5. Conclusions

This study clarified the diversity of bacterial community in pasteurized milk stored at 4 °C. Employing both culture-based methods and high-throughput sequencing, we identified predominating bacterial genera, with *Bacillus* emerging as a significant cultured bacterium. *Streptococcus* and *Acinetobacter* dominated at the genus levels within the storage period, while *Streptococcus dysgalactiae* and *Streptococcus uberis* stand out as key taxa. This study offers new evidence of the prevalence of bacterial communities in pasteurized milk, thereby adding value to the enhancement of quality control and the development of strategies for reducing microbial risks.

## Data Availability

Data is contained within the article.

## References

[1] Bosica S., Chiaverini A., De Angelis M.E., Petrini A., Averaimo D., Martino M., Rulli M., Saletti M.A., Cantelmi M.C., Ruggeri F. (2023). Severe Streptococcus equi Subspecies zooepidemicus Outbreak from Unpasteurized Dairy Product Consumption, Italy. Emerg. Infect. Dis..

[2] Porcellato D., Aspholm M., Skeie S.B., Monshaugen M., Brendehaug J., Mellegard H. (2018). Microbial diversity of consumption milk during processing and storage. Int. J. Food Microbiol..

[3] Ding R., Liu Y., Yang S., Liu Y., Shi H., Yue X., Wu R., Wu J. (2020). High-throughput sequencing provides new insights into the roles and implications of core microbiota present in pasteurized milk. Food Res. Int..

[4] Li Y., Weng P., Wu Z., Liu Y. (2023). Extending the Shelf Life of Raw Milk and Pasteurized Milk with Plantaricin FB-2. Foods.

[5] Endara P., Wiedmann M., Adalja A. (2023). Consumer willingness to pay for shelf life of high temperature, short time pasteurized fluid milk: Implications for smart labeling and food waste reduction. J. Dairy Sci..

[6] Fusco V., Chieffi D., Fanelli F., Logrieco A.F., Cho G.S., Kabisch J., Bohnlein C., Franz C. (2020). Microbial quality and safety of milk and milk products in the 21st century. Compr. Rev. Food Sci. Food Saf..

[7] Li N., Wang Y., You C., Ren J., Chen W., Zheng H., Liu Z. (2018). Variation in Raw Milk Microbiota Throughout 12 Months and the Impact of Weather Conditions. Sci. Rep..

[8] Rossvoll E., Ronning H.T., Granum P.E., Moretro T., Hjerpekjon M.R., Langsrud S. (2014). Toxin production and growth of pathogens subjected to temperature fluctuations simulating consumer handling of cold cuts. Int. J. Food Microbiol..

[9] Liu J., Zhu Y., Jay-Russell M., Lemay D.G., Mills D.A. (2020). Reservoirs of antimicrobial resistance genes in retail raw milk. Microbiome.

[10] Ranvir S., Sharma R., Gandhi K., Mann B. (2020). Assessment of physico-chemical changes in UHT milk during storage at different temperatures. J. Dairy Res..

[11] Lott T.T., Wiedmann M., Martin N.H. (2023). Shelf-life storage temperature has a considerably larger effect than high-temperature, short-time pasteurization temperature on the growth of spore-forming bacteria in fluid milk. J. Dairy Sci..

[12] Li X., Li C., Ye H., Wang Z., Wu X., Han Y., Xu B. (2019). Changes in the microbial communities in vacuum-packaged smoked bacon during storage. Food Microbiol..

[13] Bianco A., Normanno G., Capozzi L., Del Sambro L., Di Fato L., Miccolupo A., Di Taranto P., Caruso M., Petruzzi F., Ali A. (2023). High Genetic Diversity and Virulence Potential in *Bacillus cereus* sensu lato Isolated from Milk and Cheeses in Apulia Region, Southern Italy. Foods.

[14] Lan X.Y., Zhao S.G., Zheng N., Li S.L., Zhang Y.D., Liu H.M., McKillip J., Wang J.Q. (2017). Short communication: Microbiological quality of raw cow milk and its association with herd management practices in Northern China. J. Dairy Sci..

[15] Jin D., Zhao S., Zheng N., Bu D., Beckers Y., Denman S.E., McSweeney C.S., Wang J. (2017). Differences in Ureolytic Bacterial Composition between the Rumen Digesta and Rumen Wall Based on ureC Gene Classification. Front. Microbiol..

[16] Caporaso J.G., Kuczynski J., Stombaugh J., Bittinger K., Bushman F.D., Costello E.K., Fierer N., Pena A.G., Goodrich J.K., Gordon J.I. (2010). QIIME allows analysis of high-throughput community sequencing data. Nat. Methods.

[17] Edgar R.C., Haas B.J., Clemente J.C., Quince C., Knight R. (2011). UCHIME improves sensitivity and speed of chimera detection. Bioinformatics.

[18] Wang Q., Garrity G.M., Tiedje J.M., Cole J.R. (2007). Naive Bayesian classifier for rapid assignment of rRNA sequences into the new bacterial taxonomy. Appl. Environ. Microbiol..

[19] Lozupone C.A., Hamady M., Kelley S.T., Knight R. (2007). Quantitative and qualitative beta diversity measures lead to different insights into factors that structure microbial communities. Appl. Environ. Microbiol..

[20] Du B., Meng L., Wu H., Yang H., Liu H., Zheng N., Zhang Y., Zhao S., Wang J. (2022). Source Tracker Modeling Based on 16S rDNA Sequencing and Analysis of Microbial Contamination Sources for Pasteurized Milk. Front. Nutr..

[21] Edwards K.M., Badiger A., Heldman D.R., Klein M.S. (2021). Metabolomic Markers of Storage Temperature and Time in Pasteurized Milk. Metabolites.

[22] Martin N.H., Ranieri M.L., Wiedmann M., Boor K.J. (2012). Reduction of pasteurization temperature leads to lower bacterial outgrowth in pasteurized fluid milk during refrigerated storage: A case study. J. Dairy Sci..

[23] Lau S., Trmcic A., Martin N.H., Wiedmann M., Murphy S.I. (2022). Development of a Monte Carlo simulation model to predict pasteurized fluid milk spoilage due to post-pasteurization contamination with gram-negative bacteria. J. Dairy Sci..

[24] Zhai Z., Cui C., Li X., Yan J., Sun E., Wang C., Guo H., Hao Y. (2023). Prevalence, antimicrobial susceptibility, and antibiotic resistance gene transfer of *Bacillus* strains isolated from pasteurized milk. J. Dairy Sci..

[25] Gao T., Ding Y., Wu Q., Wang J., Zhang J., Yu S., Yu P., Liu C., Kong L., Feng Z. (2018). Prevalence, Virulence Genes, Antimicrobial Susceptibility, and Genetic Diversity of *Bacillus cereus* Isolated from Pasteurized Milk in China. Front. Microbiol..

[26] Zhao S., Chen J., Fei P., Feng H., Wang Y., Ali M.A., Li S., Jing H., Yang W. (2020). Prevalence, molecular characterization, and antibiotic susceptibility of *Bacillus cereus* isolated from dairy products in China. J. Dairy Sci..

[27] Porcellato D., Aspholm M., Skeie S.B., Mellegard H. (2019). Application of a novel amplicon-based sequencing approach reveals the diversity of the *Bacillus cereus* group in stored raw and pasteurized milk. Food Microbiol..

[28] Masiello S.N., Kent D., Martin N.H., Schukken Y.H., Wiedmann M., Boor K.J. (2017). Longitudinal assessment of dairy farm management practices associated with the presence of psychrotolerant Bacillales spores in bulk tank milk on 10 New York State dairy farms. J. Dairy Sci..

[29] Kobayashi T., Azuma T., Yasokawa D., Yamaki S., Yamazaki K. (2021). Spore Heat Resistance and Growth Ability at Refrigeration Temperatures of *Bacillus* spp. and *Paenibacillus* spp.. Biocontrol Sci..

[30] Oikonomou G., Addis M.F., Chassard C., Nader-Macias M.E.F., Grant I., Delbes C., Bogni C.I., Le Loir Y., Even S. (2020). Milk Microbiota: What Are We Exactly Talking About?. Front. Microbiol..

[31] Falentin H., Rault L., Nicolas A., Bouchard D.S., Lassalas J., Lamberton P., Aubry J.M., Marnet P.G., Le Loir Y., Even S. (2016). Bovine Teat Microbiome Analysis Revealed Reduced Alpha Diversity and Significant Changes in Taxonomic Profiles in Quarters with a History of Mastitis. Front. Microbiol..

[32] Xue Z., Kable M.E., Marco M.L. (2018). Impact of DNA Sequencing and Analysis Methods on 16S rRNA Gene Bacterial Community Analysis of Dairy Products. mSphere.

[33] Quigley L., O’Sullivan O., Stanton C., Beresford T.P., Ross R.P., Fitzgerald G.F., Cotter P.D. (2013). The complex microbiota of raw milk. FEMS Microbiol. Rev..

[34] Wang S., Yu Z., Wang J., Ho H., Yang Y., Fan R., Du Q., Jiang H., Han R. (2021). Prevalence, Drug Resistance, and Virulence Genes of Potential Pathogenic Bacteria in Pasteurized Milk of Chinese Fresh Milk Bar. J. Food Prot..

[35] Phuektes P., Browning G.F., Anderson G., Mansell P.D. (2003). Multiplex polymerase chain reaction as a mastitis screening test for Staphylococcus aureus, Streptococcus agalactiae, Streptococcus dysgalactiae and Streptococcus uberis in bulk milk samples. J. Dairy Res..

[36] Nakata H., Imamura Y., Saha S., Lobo R.E., Kitahara S., Araki S., Tomokiyo M., Namai F., Hiramitsu M., Inoue T. (2023). Partial Characterization and Immunomodulatory Effects of Exopolysaccharides from Streptococcus thermophilus SBC8781 during Soy Milk and Cow Milk Fermentation. Foods.

[37] McHugh A.J., Feehily C., Fenelon M.A., Gleeson D., Hill C., Cotter P.D. (2020). Tracking the Dairy Microbiota from Farm Bulk Tank to Skimmed Milk Powder. mSystems.

[38] Sun L., Lundh A., Hojer A., Bernes G., Nilsson D., Johansson M., Hetta M., Gustafsson A.H., Saeden K.H., Dicksved J. (2022). Milking system and premilking routines have a strong effect on the microbial community in bulk tank milk. J. Dairy Sci..

[39] Al-Farsi M., Al-Gharibi I., Al-Abri A., Al-Humaimi A., Al-Nabhani F., Al-Hashmi H., Al-Sarmi K., Al-Shibli S. (2021). Evaluating the shelf-life of pasteurized milk in Oman. Heliyon.

[40] Li S.N., Tang S.H., Ren R., Gong J.X., Chen Y.M. (2021). Metabolomic profile of milk fermented with Streptococcus thermophilus cocultured with Bifidobacterium animalis ssp. lactis, *Lactiplantibacillus plantarum*, or both during storage. J. Dairy Sci..

[41] Cho G.S., Li B., Rostalsky A., Fiedler G., Rosch N., Igbinosa E., Kabisch J., Bockelmann W., Hammer P., Huys G. (2018). Diversity and Antibiotic Susceptibility of Acinetobacter Strains From Milk Powder Produced in Germany. Front. Microbiol..

[42] Minst K., Martlbauer E., Miller T., Meyer C. (2012). Short communication: Streptococcus species isolated from mastitis milk samples in Germany and their resistance to antimicrobial agents. J. Dairy Sci..

[43] Crippa B.L., Rodrigues M.X., Tomazi T., Yang Y., de Oliveira Rocha L., Bicalho R.C., Silva N.C.C. (2023). Virulence factors, antimicrobial resistance and phylogeny of bovine mastitis-associated Streptococcus dysgalactiae. J. Dairy Res..

[44] Wente N., Kromker V. (2020). Streptococcus dysgalactiae-Contagious or Environmental?. Animals.

[45] Lan R., Zhou Y., Wang Z., Fu S., Gao Y., Gao X., Zhang J., Han X., Phouthapane V., Xu Y. (2022). Reduction of ROS-HIF1alpha-driven glycolysis by taurine alleviates Streptococcus uberis infection. Food Funct..

[46] Mihklepp K., Kivirand K., Juronen D., Lookene A., Rinken T. (2019). Immunodetection of Streptococcus uberis pathogen in raw milk. Enzyme Microb. Technol..

[47] Achek R., El-Adawy H., Hotzel H., Tomaso H., Ehricht R., Hamdi T.M., Azzi O., Monecke S. (2020). Short communication: Diversity of staphylococci isolated from sheep mastitis in northern Algeria. J. Dairy Sci..

